# Nasopharyngeal Bacterial Prevalence and Microbial Diversity at First Treatment for Bovine Respiratory Disease (BRD) and Its Associations with Health and Mortality Outcomes in Feedyard Cattle

**DOI:** 10.3390/microorganisms12010033

**Published:** 2023-12-24

**Authors:** Kyndall Neal, Raghavendra G. Amachawadi, Brad J. White, Teresa D. Shippy, Miles E. Theurer, Robert L. Larson, Brian V. Lubbers, Michael Kleinhenz

**Affiliations:** 1 Beef Cattle Institute, Department of Clinical Sciences, College of Veterinary Medicine, Kansas State University, Manhattan, KS 66506, USA; knorris34@vet.ksu.edu (K.N.); bwhite@vet.ksu.edu (B.J.W.); rlarson@vet.ksu.edu (R.L.L.); blubbers@vet.ksu.edu (B.V.L.); 2 Data Science Center, Division of Biology, Kansas State University, Manhattan, KS 66506, USA; tshippy@ksu.edu; 3 Veterinary Research and Consulting Services LLC, Hays, KS 67601, USA; miles@vrcsllc.com; 4 Veterinary Education, Research and Outreach, Texas A&M University, Canyon, TX 79015, USA; mkleinhenz@tamu.edu

**Keywords:** bovine respiratory disease, cattle, first treatment, health outcomes, nasopharyngeal microbiota

## Abstract

Bovine respiratory disease (BRD) is an economically important disease in feedyards influencing both animal welfare and antimicrobial utilization. Major pathogens associated with BRD have been identified in previous research, but little information is available on the relationship between nasopharyngeal microbiota and health outcomes. The objective of this study was to identify potential associations between nasopharyngeal microbiota and antimicrobial resistance patterns of clinical cases that lived or died compared to non-diseased controls. Enrolled animals were subdivided based on clinical disease status and case outcome (subsequent mortality). Deep nasopharyngeal swabs were collected on enrolled animals and submitted for bacterial isolation, antimicrobial susceptibility determination, and metagenomics analysis. Enrolled cattle were represented in three groups: animals at first treatment for BRD that subsequently died (BRDM, *n* = 9), animals at first treatment for BRD that subsequently lived (BRDL, *n* = 15), and animals that were never treated for BRD during the feeding phase (CONT, *n* = 11). Antimicrobial resistance patterns for *Pasteurella multocida* illustrated cattle in each outcome category had isolates that were pan-susceptible or only showed resistance to oxytetracycline. The relative abundance of species and genera illustrated few differences among the three outcomes. Higher alpha diversity was identified in BRDL compared to CONT at the species level, and both BRDL and BRDM showed increased alpha diversity compared to CONT at the general level. Overall, this work illustrated nasopharyngeal microbiota showed relatively few differences among BRD cases that lived or died compared to animals without BRD.

## 1. Introduction

Bovine respiratory disease (BRD) is a major cause of morbidity and mortality in beef cattle feedyards [1,2,3,4,5]. This syndrome can have significant economic impacts on feedyards by lowering an animal’s average daily gain, thus increasing feed and housing costs associated with that animal [6,7,8]. Economically, mortality is severe as it results in a total loss of all resources invested in that animal. Thus, understanding factors that could result in fatality following treatment for disease is important [9]. While common pathogens associated with BRD cases have been identified, little research has been done evaluating differences in case outcomes based on deep nasopharyngeal microbiota at the time of treatment [10,11,12].

Various factors can predispose cattle to BRD, including host, environmental, and agent factors. Some of the BRD causative agents include *Mannheimia haemolytica*, *Histophilus somni*, *Mycoplasma bovis*, and *Pasteurella multocida*. Many different pathogens play a role in the development of BRD, which creates complex interactions among microbial populations [13]. Other bacteria, which may or may not be associated with BRD, commonly found in nasopharyngeal samples of cattle, both healthy and with respiratory disease, include the genera *Moraxella*, *Acinetobacter*, *Corynebacterium*, *Clostridium*, *Solibacillus*, *Turicibacter*, *Bacteroides*, and *Blautia.* In one study, the most prevalent bacteria isolated from nasopharyngeal samples taken upon entry into the feedyard belonged to the *Mycoplasma*, *Moraxella*, and *Acinetobacter* genera [14]. However, it was observed that cattle with clinical BRD had a higher prevalence of *Acinetobacter*, *Solibacillus*, and *Pasteurella* compared to clinically healthy cattle. Furthermore, even though *Mannheimia* is commonly recognized as a part of the BRD complex, there was no observed increase in the relative prevalence of *Mannheimia* in BRD-affected cattle compared to healthy cattle [15]. While the prevalence of bacteria in the nasopharynx has been studied, little work has been done evaluating outcomes associated with different spectrums of nasopharyngeal microbial populations.

Understanding the exact relationship between BRD and the nasopharyngeal microbiome requires more research as it is difficult to ascertain whether the microbiome at the time of sampling leads to clinical BRD cases or if the immune system response to clinical BRD, in turn, alters the microbiome [16]. Studies of cattle at the time of the first BRD treatment in relation to bacterial prevalence, antimicrobial resistance, and nasopharyngeal microbiome are sparse. The objective of this study was to identify potential associations between nasopharyngeal microbiota and antimicrobial resistance patterns in clinical cases that lived or died compared to non-diseased controls.

## 2. Materials and Methods

All research and procedures were approved prior to trial initiation by the Kansas State University Institutional Animal Care and Use Committee (IACUC#4588. 99).

### 2.1. Animals and Nasopharyngeal Swab Collection

Deep nasopharyngeal swabs (2 per animal) were collected from cattle located at a collaborating feedyard in southwest Kansas at the time of first treatment for BRD and from a non-BRD counterpart for comparison. Individual animals were identified as potential BRD cases by evaluation of clinical signs in the housing pen (depression, anorexia, increased respiration), then further evaluated in the treatment chute (rectal temperature). Non-diseased cases were selected as cattle not showing clinical signs of BRD and not subsequently treated for BRD during the rest of the feeding phase. Each BRD case was monitored to determine post-treatment mortality during the feeding phase. Samples were collected in a manner to create three cattle groups for comparison: cattle treated for BRD with subsequent mortality (BRDM), cattle treated for BRD with no subsequent mortality (BRDL), and a control group of cattle not treated for BRD (CONT).

Swabs were placed in 2 mL cryovials, labeled with the animal number, and stored immediately in liquid nitrogen until shipped to Kansas State University. On arrival, swab samples were stored in a −80 °C freezer until analysis. Following the collection of all samples, subsets of samples were created, including first-treatment BRD cases that resulted in fatality, first-treatment BRD cases that did not result in fatality, and non-BRD case comparisons.

### 2.2. Bacterial Isolation and Identification

From the pooled nasopharyngeal swab samples, the isolation of *Pasteurella* spp. and *M. haemolytica* was carried out as described, respectively [17,18]. Two isolates of each nasopharyngeal pathogen exhibiting a typical colony morphology were subjected to catalase and oxidase biochemical tests. Genus and species confirmation of *P. multocida* and *M. haemolytica* was done by PCR [19]. The confirmed isolates (two isolates of each species per sample) were stored in Cryocare-protect^®^ beads (Key Scientific Products, Stamford, TX, USA) at −80 °C for future use.

### 2.3. Antimicrobial Susceptibility Determinations

Minimal inhibitory concentrations (MIC) were measured with the broth-microdilution method according to CLSI guidelines (2023) using the Sensititre^®^ automated antimicrobial system (Trek Diagnostics Systems, Cleveland, OH, USA). A commercially available Bovine Tulathromycin MIC format Sensititre^®^ (BOPO-7F) panel plate was used in conjunction with the Sensititre^®^ automated inoculation delivery system (Trek Diagnostics Systems, Cleveland, OH, USA). Appropriate ATCC (American Type Culture Collection, Manassas, VA, USA) quality control strains, specifically *Escherichia coli* ATCC 25922 and *Enterococcus faecalis* ATCC 29212, served as reference standards in conducting susceptibility testing. The MIC for each isolate was documented and categorized as either resistant, intermediate, or sensitive in accordance with established guidelines [20].

### 2.4. DNA Extraction from Nasopharyngeal Samples

Total DNA was extracted from the pooled nasopharyngeal samples using the “PowerSoil^®^ DNA isolation kit” (MO BIO Laboratories; Carlsbad, CA, USA) according to the manufacturer’s protocol. The isolated DNA was stored at −20 °C until used for metagenome shotgun sequencing.

### 2.5. Library Preparation and Sequencing

Quantity and quality of genomic DNA (gDNA) samples were determined with high sensitivity dsDNA Qubit assays and Agilent TapeStation gel analysis, respectively. Construction of sequencing libraries was performed using the Illumina DNA Prep sequencing library kit (Illumina, San Diego, CA, USA) with initial amounts of gDNA ranging from 288–500 ng. During library construction, gDNA was subjected to bead-based tagmentation to fragment the DNA, add adapter sequences, and normalize library concentrations. Fragments were then amplified with five cycles of PCR to incorporate unique dual-index adapters. Quality control on the libraries was performed with Qubit and TapeStation assays. Library preps were pooled by ng amount, and concentration was confirmed via Illumina Library Quant qPCR assay (Roche, Indianapolis, IN, USA). Paired-end 150-base sequencing reads were generated using an Illumina NextSeq2000 system at the University of Kansas Genome Sequencing Core. Base call files were produced by the instrument’s Real Time Analysis software and then demultiplexed and converted to FASTQ files using DRAGEN BCL Convert software (Version 2.20).

### 2.6. Bioinformatics Analysis

Software for sequence analysis was installed and run on Beocat, the Kansas State University High-Performance Computing Cluster. Using the AMRPlusPlus v2.0 pipeline [21], raw reads were subjected to adapter and quality trimming with Trimmomatic 0.39 [22]. These trimmed reads were aligned to the *Bos taurus* reference genome (GCF_002263795.2_ARS-UCD1.3_genomic.fna.gz) plus the Y chromosome sequence (Bos_taurus_Y_CM001061.2. fasta) using Burrows-Wheeler Alignment (BWA) [23]. More than 99% of reads aligned to the host genome and were removed from further analysis using BEDTools [24]. The taxonomic classification of the remaining non-host reads was assigned using Kraken 2 [25]. The identified non-host reads were then processed using the updated resistome analysis features of AMRPlusPlus v3.0 [26] with the MEGARes v3.0 database, as well as with ARIBA [27] using the CARD database [28] to identify reads mapping to potential Antimicrobial Resistance (AMR) genes and to check for SNPs in those reads.

Statistical analysis was performed in R (R Core Team, 2022) using species-level read counts produced by Kraken2. Linear models (using the lm function with anova from the R stats package) were used to test for the significant effect of group (CONT, BRDM, BRDL) on raw read counts and non-host read counts. Post hoc pairwise testing was performed using the TukeyHSD function. Before normalization and additional analysis, the raw read counts were filtered to keep only bacterial taxa with at least 10 total counts across all samples. Counts were then normalized by the Cumulative Sum Scaling method using the cumNorm function of metagenomeSeq [29]. The tax_glom function of phyloseq [30] was used to aggregate the normalized counts to the species, genus, and phylum levels.

Relative taxa abundance by group was calculated at the species, genus, and phylum levels using normalized, aggregated counts. For the species and genus levels, taxa with relative abundance less than 1% were grouped together as “low abundance” taxa. Relative Abundance bar plots were generated with the geom_bar function of ggplot2 [31]. The estimate_richness function of phyloseq was used to calculate alpha diversity for species, genus, and phylum levels. The Observed, Shannon, and Inverse Simpson indices were compared between groups using ANOVA of linear models (lm function) to test for significant differences. The TukeyHSD function was used for post hoc pairwise testing. The ordinate function of the vegan [32] package was used to create non-metric multidimensional scaling (NMDS) plots based on the Bray-Curtis distance. The stat_ellipse function of ggplot2 was used to add ellipses showing 95% confidence intervals for a multivariate t-distribution. PERMANOVA testing [33] via the adonis function of vegan was used to check for significant differences in beta diversity between sample groups. The ANCOMBC package [34,35] was used to test for significant differences in species and genus abundance between sample groups with the default Holm-Bonferroni method used for multiple testing correction. Before heatmap creation, additional filtering was applied with the phyloseq_filter_taxa_rel_abund function of metagMisc [36] to remove species with relative abundance less than 0.005. Aggregated counts for species with relative abundance greater than 0.005 were used to create a heatmap using the plot_heatmap function of phyloseq. The ordering of rows (species) was determined by NMDS ordination, and columns (samples) were ordered by group. All Illumina sequence data from this study have been deposited in the NCBI’s BioProject ID PRJNA1047266.

## 3. Results

### 3.1. Bacterial Prevalence

A total of 99 samples were collected for potential participation in the study. The BRDM group (n = 9) consisted of cases sampled at first treatment for BRD that subsequently died. Samples for the BRDL group were collected from cattle that did not die at the time of first treatment for BRD (n = 76). Additional cattle not treated for BRD during their feeding period were sampled as CONT (n = 11). Due to resource allocation, only limited samples were submitted for culture, susceptibility testing, and sequencing procedures. Final submissions included all BRDM (n = 9) cases and randomly selected samples from BRDL (n = 15) and CONT (n = 11). Of the 35 samples tested, only one sample (from the BRDL group) was positive on culture and confirmed by PCR for *M. haemolytica*. The sole isolated sample of *M. haemolytica* was susceptible to all antimicrobials except penicillin, to which it was resistant. Nine of our 35 samples were positive via culture and confirmed by PCR for *P. multocida*, and this was distributed among groups: CONT (n = 3), BRDL (n = 4), and BRDM (n = 2).

### 3.2. Antimicrobial Minimal Inhibitory Concentrations

The nine isolates of *P. multocida* showed varying drug resistance in Table 1. The bacteria from Sample 1 (BRDM) and Sample 2 (BRDL) were susceptible to all antimicrobials. Sample 3 (CONT) and sample 4 (BRDL) produced isolates with intermediate susceptibility to penicillin and susceptibility to all other antimicrobials tested. *P. multocida* isolated from sample 5 (BRDL) was resistant to oxytetracycline but susceptible to all others. Isolates from samples 6 and 7 (CONT) showed mild resistance to oxytetracycline, intermediate susceptibility to chlortetracycline, and susceptibility to all other antimicrobials. Sample 8 (BRDM) contained *P. multocida*, which is resistant to oxytetracycline, intermediate to spectinomycin, and susceptible to all other antimicrobials. *P. multocida* from sample 9 (BRDL) was resistant to oxytetracycline, intermediate to penicillin, and susceptible to all other antimicrobials.

### 3.3. Shotgun Metagenomics

**Figure 1:**
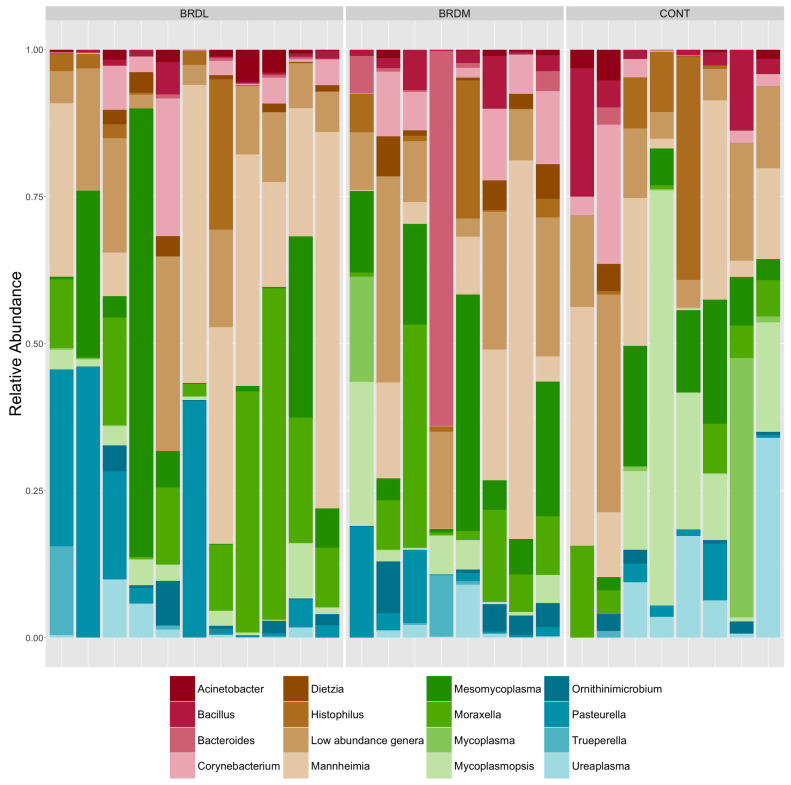
Genera relative abundance in samples grouped by Classification group (CONT (non-BRD cattle), BRDL (cattle treated for BRD that lived), and BRDM (cattle treated for BRD that died). Genera relative abundance under one percent are grouped as Low abundance taxa.

**Figure 2:**
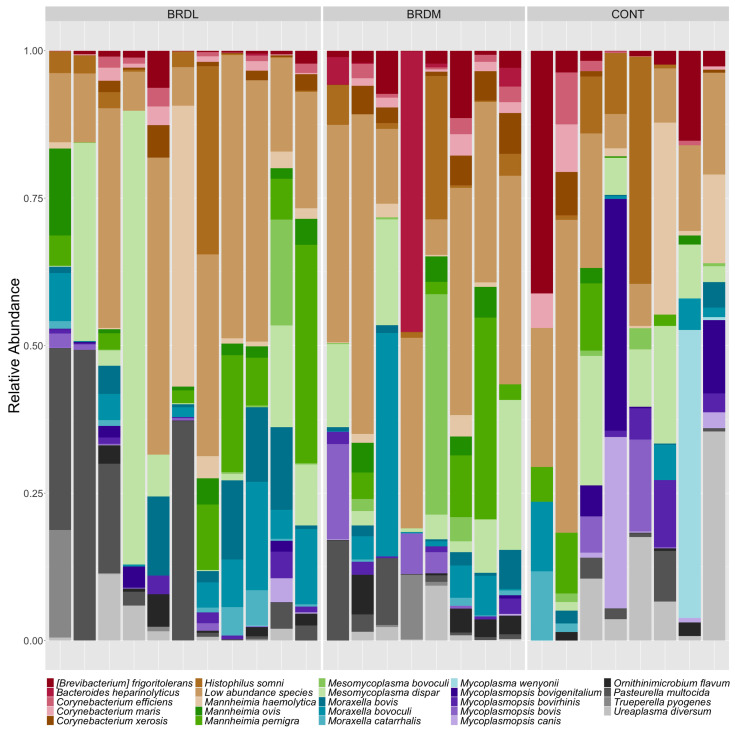
Species relative abundance in samples grouped by Classification group (CONT (non-BRD cattle), BRDL (cattle treated for BRD that lived), and BRDM (cattle treated for BRD that died). Species with relative abundance under one percent are grouped as Low abundance taxa.

Of the 35 samples, 8 samples did not yield enough DNA for further testing. We obtained shotgun metagenomic sequences from 27 samples (which were assigned sample IDs 91-117 for sequencing). Of these, eight were CONT samples, 11 were from animals that survived BRD (BRDL), and eight were from animals that died (BRDM). A total of 670.9 million reads (335.45 million paired reads) were sequenced, with individual sample counts ranging from 16,152,088 to 29,512,202, with a median of 26,260,584 counts. Significantly fewer raw reads were obtained from the CONT samples than from the BRDL and BRDM samples. Less than 1% of reads were removed because of low quality, but over 99% of the remaining reads were bovine host reads, which were removed from further analysis. Non-host read counts ranged from 6408 to 97,130 reads per sample, with an average of 22,510 reads. Despite the difference in raw reads between sample groups, the difference in non-host read counts between sample groups was not significant. Non-host reads were taxonomically classified with Kraken 2 [25] within the AMRPlusPlus v2.0 pipeline [21]. After the removal of non-bacterial and very low abundance taxa, 328 taxa remained, of which 252 were classified to the species level. These taxa represented 96 genera and 6 phyla. We calculated the relative abundance of taxa in each sample and used stacked bar graphs to visualize the results of Figure 1 (genus) and Figure 2 (species). No species or genera showed statistically significant differences in relative abundance between the CONT, BRDM, and BRDL groups. After ANCOMBC2 [34,35] global testing with Holm-Bonferroni correction for multiple comparisons, all taxa had a q value of 1.

Although we found no significant difference in the relative abundance of individual species between groups, we decided to look more closely at the prevalence of four bacteria commonly associated with BRD: *Histophilus somni*, *Mannheimia hemolytica*, *Mycoplasmosis bovis*, and *Pasteurella multocida*. With respect to the raw read count number for each of these species, at least one read classified as *M. haemolytica* was present in 21 of the 27 samples, while 23 had at least one read classified as *P. multocida*. The samples with the highest counts of *P. multicide* and/or *M. haemolytica* were all in the BRDL group of samples. At least one read was classified as *H. somni* in 22 of the 27 samples. Samples with some *H. somni* reads were found in all groups, but none had extremely high counts. *M. bovis* was represented by at least one read in 15 of the 27 samples, but like *H. somni,* did not have high counts in any samples and was found in a few samples in all three groups.

**Figure 3:**
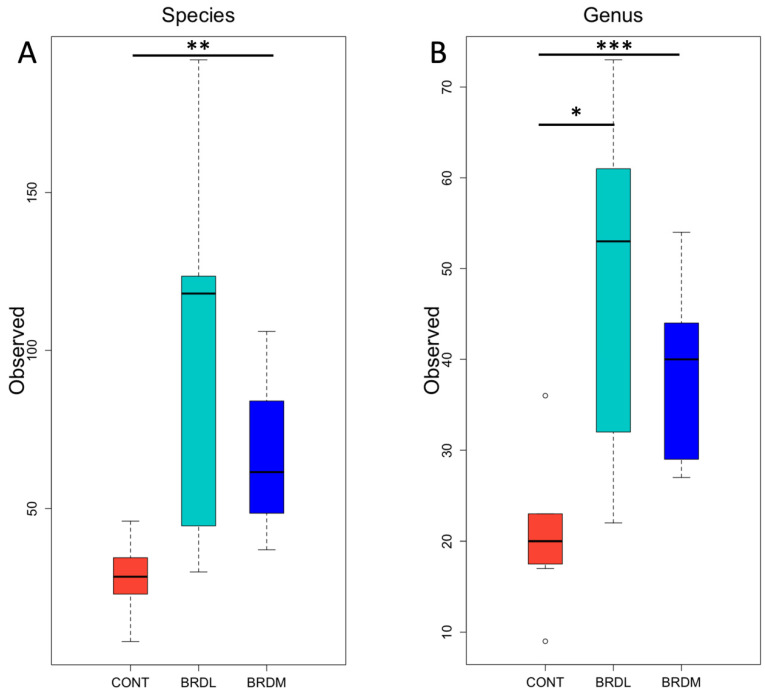
Observed (richness) metric of alpha diversity at (**A**) species level and (**B**) genus level for CONT (non-BRD cattle), BRDL (cattle treated for BRD that lived), and BRDM (cattle treated for BRD that died) groups. The *Y* axis shows the number of taxa present. Boxes denote the 25th to 75th percentile (interquartile range) for the group, with the horizontal line within the box indicating the median value. Post hoc pairwise comparisons with significant adjusted *p* values are indicated by horizontal lines with asterisks (* = <0.05, ** = <0.01 and *** = <0.001).

**Figure 4:**
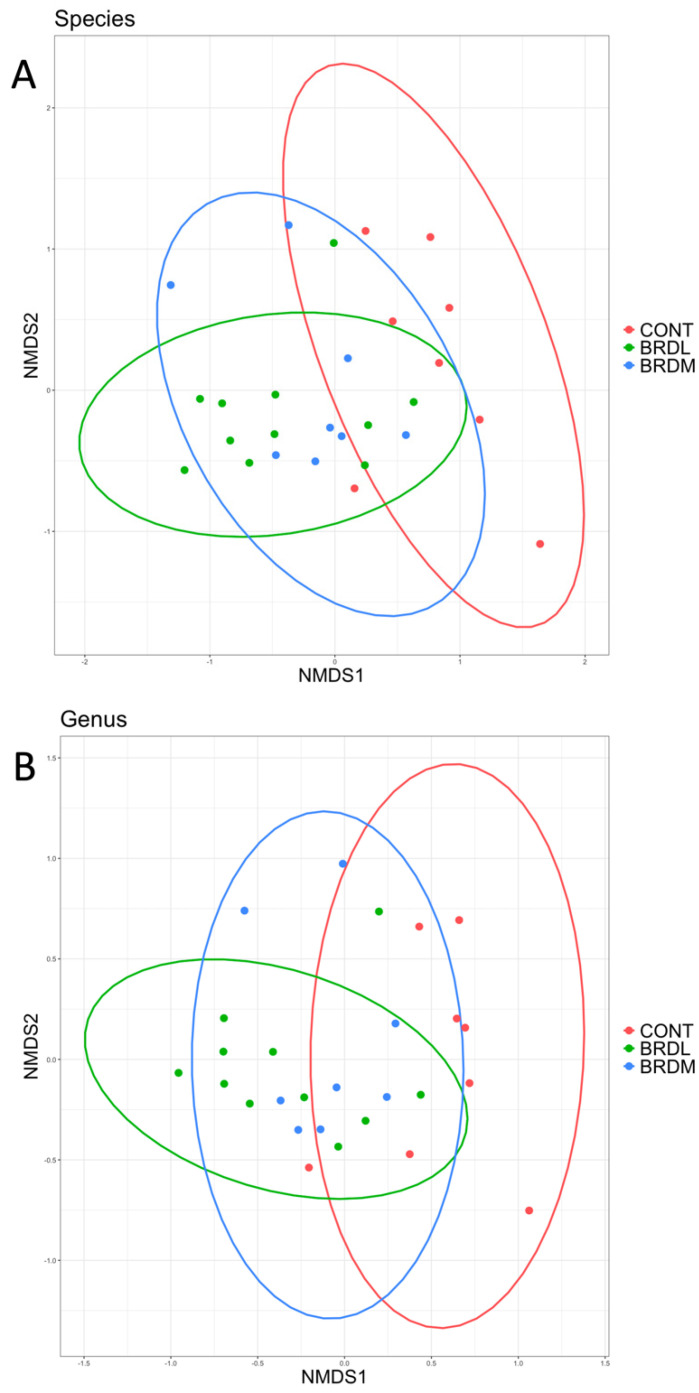
NMDS plot of beta diversity at the (**A**) species and (**B**) genus level. No significant differences were found between sample groups: CONT (non-BRD cattle), BRDL (cattle treated for BRD that lived), and BRDM (cattle treated for BRD that died).

Alpha diversity for each sample was measured at the species and genus levels by calculating Observed, Shannon, and Inverse Simpson indices. The observed (richness) metric was significantly higher in BRDM samples than in the CONT samples at the species level and in both BRDL and BRDM samples compared to CONT samples at the genus level in Figure 3. We visualized beta diversity using NMDS plots of Figure 4 and used PERMANOVA testing [33] to assess the statistical significance of differences between our sample groups. We found no significant differences in beta diversity between sample groups at either the species or genus level.

### 3.4. AMR Genes

To search for antimicrobial resistance (AMR) genes in the shotgun metagenomic sequences, we used AMRPlusPlus v3.0 [26], which features an updated resistome analysis pipeline, and ARIBA [27] to map non-host reads identified by AMRPlusPlus v2.0 to known AMR genes. AMRPlusPlus v3.0 identified eight potential AMR genes across all samples, but only five of these were found to carry sequences known to be associated with AMR, as shown in Table 2. Three samples, one CONT (94) and two BRDL (102 and 111), had at least one of the verified AMR alleles.

## 4. Discussion

Samples were evaluated from cattle based on outcomes following BRD treatment (BRDM, BRDL) or cattle never treated for BRD (CONT) to determine potential differences in bacterial profiles, antimicrobial resistance patterns, the relative abundance of species/genera, and alpha and beta diversity indices. Common BRD pathogens were identified with relatively minor changes in antimicrobial susceptibility among cattle outcome classification groups. The relative abundance of species and genera did not illustrate large visual differences among the outcome groups; however, the alpha diversity index illustrated differences between BRD-treated animals (BRDM and BRDL) compared to cattle never treated (CONT).

*Mannheimia haemolytica* is often reported as a primary pathogen identified in fatal BRD cases but was rarely identified by culture in this study [37,38]. *Mannheimia haemolytica* is not normally considered a zoonotic agent; this can induce severe illnesses in human infants and immunocompromised adults. Sporadically, this pathogen has been observed in cases of septicemia among infants and heart disease in adults [39]. *Pasteurella multocida* was cultured from 9 samples, allowing for antimicrobial susceptibility testing. While not enough numbers of cattle were present in each category for statistical analysis, cattle in each outcome category had isolates either pan-susceptible or only showing resistance to oxytetracycline. In BRDM, one isolate was pan-susceptible, and one illustrated oxytetracycline resistance. Similarly, BRDL had two isolates that were pan-susceptible and two isolates that were resistant to oxytetracycline. The CONT cattle had a similar ratio, with one isolate pan-susceptible and two resistant to oxytetracycline. No resistance to commonly used macrolides was identified, and each outcome category had animals possessing both susceptible and resistant isolates. This finding is logical as samples were collected prior to the BRD antimicrobial treatment, and resistance patterns may have been influenced by previous antimicrobial treatment programs, which would be similar among cattle in all three groups. Few samples had known AMR genes identified with shotgun metagenomics: only three animals (1 CONT and 2 BRDL) were able to be evaluated. In this small study, differences in antimicrobial resistance patterns were not identified in BRD cases that lived or died. Zoonotic transmission of *Pasteurella multocida* to humans typically occurs through animal bites or contact with nasal secretions. Common symptoms of *P. multocida* infections in humans include swelling, cellulitis, and the presence of bloody or purulent exudate at the site of the wound [40]. 

The relative abundance of species and genera were compared among all three outcomes. Cattle in each class showed the presence of common pathogenic (*Mannheimia haemolytica*, *Pastuerella multocida*, *Histophilus somni*, and *Mycoplasma*) species. Finding *Mannheimia* species and genera on shotgun metagenomics but not on culture is not surprising due to the increased sensitivity of the metagenomics testing compared to culture. Few major differences were identified visually or statistically using relative abundance to compare BRDM, BRDL, and CONT groups. This finding is consistent with previous research comparing BRD-treated animals to non-BRD-treated animals [41]. McMullen et al. noted more changes in bacterial populations over time compared to bacterial population differences among BRD and non-BRD cattle [41].

The alpha diversity was higher in BRDL compared to CONT at the species level, and both BRDL and BRDM showed higher alpha diversity compared to CONT at the genus level. While differences in alpha diversity were identified relative to CONT cattle, no differences were found among the outcomes of BRD-treated cattle. Increased alpha diversity in cattle treated for BRD differs from previous research that illustrated higher species richness and Shannon diversity in cattle not treated for BRD. One potential difference is in the previous study, cattle raised without antimicrobials were used as the study population, and in the current study, cattle could have received previous antimicrobial treatment [42]. Another study identified less diverse bacterial species in cattle that died of BRD [43]; however, this study evaluated cattle with previous treatments, and in the current study, cattle were evaluated at first treatment for BRD. Antimicrobial treatment would likely impact the diversity of bacterial species.

Limitations of this study include a relatively small sample size from each outcome (BRDL, BRDM, and CONT) submitted for full testing; however, relatively few differences were identified in the evaluations. However, we believe our study makes a valuable contribution to the field of BRD and highlights the importance of differences in the microbiota composition among treated versus control animals. Moreover, this is a small pilot study conducted to generate preliminary data for the potential follow-up of a large longitudinal study in a commercial feedlot. Cattle enrolled in the study were from a commercial facility, and no history prior to arrival at the facility was available; therefore, previous antimicrobial or disease treatments could not be included in the analysis.

Results from this study illustrated relatively few major differences in bacterial populations, antimicrobial resistance patterns, or relative abundance among cattle treated for BRD that lived, cattle treated for BRD that died, or animals never treated for BRD. The alpha diversity index was higher at both species and genera level for BRD-treated animals compared to CONT. While some differences were identified relative to CONT cattle, no apparent differences were identified between BRDL and BRDM groups.

## Figures and Tables

**Table 1:** Antimicrobial susceptibilities of *Pasteurella multocida* isolated from first-treatment bovine respiratory disease (BRD) cattle in comparison to healthy cattle. Samples are labeled with classification group for study: CONT (non-BRD cattle), BRDL (cattle treated for BRD that lived), and BRDM (cattle treated for BRD that died).

Antimicrobial	MIC Susceptible	MIC Intermediate	MIC Resistant	Sample 1 BRDM	Sample 2 BRDL	Sample 3 CONT	Sample 4 BRDL	Sample 5 BRDL	Sample 6 CONT	Sample 7 CONT	Sample 8 BRDM	Sample 9 BRDL
Ampicillin	≤0.03	0.06–0.12	≥0.25	<0.25	<0.25	<0.25	<0.25	<0.25	<0.25	<0.25	<0.25	<0.25
Ceftiofur	≤2	4	≥8	<0.25	<0.25	<0.25	<0.25	<0.25	<0.25	<0.25	<0.25	<0.25
Chlortetracycline	≤2	4	≥8	2	<0.5	<0.5	<0.5	1	4	4	2	2
Danofloxacin	≤0.25	0.5	≥1	<0.12	<0.12	<0.12	<0.12	<0.12	<0.12	0.25	<0.12	<0.12
Enrofloxacin	≤0.25	0.5–1	≥2	<0.12	<0.12	<0.12	<0.12	<0.12	<0.12	<0.12	<0.12	<0.12
Florfenicol	≤2	4	≥8	<0.25	0.5	1	1	0.5	0.5	0.5	0.5	1
Oxytetracycline	≤2	4	≥8	<0.5	<0.5	<0.5	<0.5	>8	>8	>8	>8	>8
Penicillin	≤0.25	0.5	≥1	<0.12	<0.12	0.5	0.5	<0.12	<0.12	0.25	<0.12	0.5
Spectinomycin	≤32	64	≥128	<8	16	32	32	16	16	32	>64	32
Tulathromycin	≤16	32	≥64	2	<1	16	16	<1	2	16	<1	2

**Table 2:** Presence of antimicrobial resistance genes in total community DNA isolated from nasopharyngeal swabs from a first treatment bovine respiratory disease (BRD) cattle in comparison to healthy cattle.

MEGARes Database Accession	Sequenced Samples					
	94 CONT	102 BRDL	111 BRDL	112 BRDL	113 BRDL	114 BRDL
MEG_2860|Drugs|MLS| 23S_rRNA_methyltransferases|ERMX	0	13/13	0	0	0	0
MEG_3977|Drugs|MLS| Macrolide-resistant_23S_rRNA_mutation|MLS23S| RequiresSNPConfirmation	0	0	0/341	0	0	0
MEG_3978|Drugs|MLS| Macrolide-resistant_23S_rRNA_mutation MLS23S|RequiresSNPConfirmation	0	0/219	0	0	0/290	0/64
MEG_3979|Drugs|MLS| Macrolide-resistant_23S_rRNA_mutation|MLS23S |RequiresSNPConfirmation	0	17/158	0	0	0	0
MEG_3983|Drugs|MLS| Macrolide-resistant_23S_rRNA_mutation|MLS23S| RequiresSNPConfirmation	4/37	0	0	0	0	0
MEG_6|Drugs|Aminoglycosides| Aminoglycoside-resistant_16S_ribosomal_subunit_protein|A16S| RequiresSNPConfirmation	0	0/319	0/401	0/36	0/63	0
MEG_7310|Drugs|Elfamycins|EF-Tu_inhibition|TUFAB|RequiresSNPConfirmation	0	4/55	11/102	0	0	0
MEG_8249|Drugs|MLS| Macrolide-resistant_23S_rRNA_mutation|MLS23S| RequiresSNPConfirmation	0	196/196	248/248	0	0	0

For each potential AMR gene identified by AMRPlusPlus v3.0 in at least one sample, counts of AMR-verified reads are given as the numerator, and total read counts for that gene are the denominator.

## Data Availability

Data available from the corresponding author upon reasonable request.
